# Radial arterial versus left ventricular dP/dt for assessment of contractility in patients undergoing transcatheter aortic valve replacement: a retrospective observational study

**DOI:** 10.1186/s12872-026-06159-z

**Published:** 2026-06-25

**Authors:** Steven Garcia, Ellie Tsang, Jacqueline Chen, Neal W. Fleming

**Affiliations:** 1 Sutter Roseville Medical Center, Roseville, CA 95663 United States; 2 MS4, UC Davis School of Medicine, Sacramento, CA 95817 United States; 3 G2 Anesthesia, Los Gatos, CA 95032 United States; 4 Department of Anesthesiology & Pain Medicine, University of California, Davis School of Medicine, Sacramento, CA United States; 5 Department of Anesthesiology & Pain Medicine, UC Davis Medical Center, 4150 V Street PSSB – Suite1200, Sacramento, CA 95817-1460 United States

**Keywords:** Contractility, dP/dt, Transcatheter Aortic Valve Replacement (TAVR)

## Abstract

**Background:**

Left ventricular (LV) contractility is a core characterization of cardiac function that provides therapeutic guidance in both chronic and acute patient care. Measurement of the change in pressure over time in the left ventricle (LV dP/dt) is a clinical gold standard for evaluating cardiac contractility but is invasive and has associated risks. Measurement of dP/dt from a radial arterial pressure catheter offers a less invasive and safer alternative. Studies comparing these two measurements are limited and have conflicting conclusions. The objective of this study was to evaluate the correlations between LV and radial arterial dP/dt and clarify the clinical utility of radial dP/dt.

**Methods:**

This was a retrospective observational study carried out at a large tertiary academic medical center. Data was collected from the electronic medical records of patients who underwent transcatheter aortic valve replacement (TAVR) with an Edwards Acumen IQ transducer attached to the radial arterial catheter to measure dP/dt. Concurrent measurements of dP/dt were recorded from LV and radial arterial catheters before and after TAVR. Comparisons included Bland-Altman analysis, concordance changes following TAVR, Spearman correlations and linear regression analysis.

**Results:**

Bland-Altman analysis before TAVR demonstrated a bias between LV and radial measurements of 621 ± 396 mmHg/s with 95% limits of agreement from − 155 to 1397 mmHg/s. After TAVR, the bias decreased to 54 ± 412 mmHg/s, with 95% limits of agreement from − 754 to 862 mmHg/s. Concordance analysis of the changes following TAVR demonstrated an inverse relationship with a decrease in LV dP/dt and an increase in radial dP/dt observed in 73% of the patients. Correlation analysis before TAVR revealed Spearman *r* = 0.16 (95% CI: -0.06,0.37). After TAVR there was no significant change in the correlation coefficient, Spearman *r* = 0.09 (95% CI: -0.14, 0.31), but the slope of the best-fit regression line increased from 0.48 (95% CI: 0.44, 0.53) to 0.88 (95%CI: 0.80, 0.97).

**Conclusion:**

Severe aortic stenosis impacts the relationship between LV and radial measurements of dP/dt. Following TAVR the correlation remains poor, but linear regression analysis suggests radial measurement of dP/dt may have the clinical utility to characterize directional changes of contractility within an individual patient.

**Trial Registration:**

Clinical trial Number: not applicable. The study protocol was reviewed by the institutional human subjects research committee, which waived the need for written, informed consent. As a quality improvement project designed to evaluate the clinical utility of radial arterial dP/dt measurement it was not registered on the ClinicalTrials.gov website.

**Supplementary Information:**

The online version contains supplementary material available at https://doi.org/10.1186/s12872-026-06159-z.

## Background

Left ventricular (LV) contractility is a core characterization of cardiac function as it assesses the heart’s ability to pump blood under specific preload and afterload conditions. Measurements of contractility provide therapeutic guidance in both chronic and acute patient care. Direct measurement of the change in pressure over time in the left ventricle (LV dP/dt) is a clinical gold standard for evaluating cardiac contractility [1, 2]. While objectively accurate, measuring LV dP/dt is invasive, requiring catheterization of the left ventricle and associated risks including perforation, embolization and dysrhythmias.

Although there are differences in waveform morphology, measurement of dP/dt from a radial arterial pressure catheter waveform offers a less invasive and more accessible alternative for measuring cardiac contractility. Some studies have found that radial arterial dP/dt correlates strongly with LV dP/dt [3, 4] while others have not confirmed this [5]. Due to the minimal and conflicting literature, the objective of this study was to evaluate the correlations between LV and radial arterial dP/dt and clarify the clinical utility of radial dP/dt.

## Methods

### Patient selection and enrollment

This retrospective observational study protocol was reviewed by the University of California, Davis human subjects research committee through their self-certification pathway which characterized the protocol as quality improvement and waved the need for written, informed consent. As a quality improvement project designed to evaluate the clinical utility of radial arterial dP/dt measurement it was not registered on the ClinicalTrials.gov website so a Clinical trial number is not applicable. All patients who underwent transcatheter aortic valve replacement (TAVR) at a single large tertiary academic medical center and had both LV dP/dt and radial arterial dP/dt (Acumen IQ sensor, HemoSphere monitor, Edwards LifeSciences, Irvine, CA) measurements were included in the study.

### Data collection

As standard protocol for TAVR, left ventricular pressures including maximum dP/dt were routinely recorded before and after valve deployment using a 6Fr pigtail catheter (Cordis, Miami Lakes, FL) and calculated using the Mac-Lab hemodynamic monitoring system (GE Healthcare, Chicago, IL). The LV dP/dt values were obtained retrospectively from the TAVR procedure report. The number of measurements available at each time point varied between one and three and were averaged for final data analysis. The corresponding radial arterial pressure and dP/dt values were obtained from the electronic anesthetic record which recorded all hemodynamic parameters calculated from the HemoSphere monitoring platform (Edwards LifeSciences, Irvine, CA), at one-minute intervals. Beat-to-beat synchronization was not possible, so time-synchronized values were averaged for the final data analysis. Radial pressures were recorded using an 20ga, 4.5 cm catheter (Arrow, Teleflex, Wayne, PA) and an Edwards Acumen IQ transducer (Edwards LifeSciences, Irvine, CA). For the pre-aortic valve deployment values, the radial arterial dP/dt was a three-minute average of the HemoSphere measurements ending with the LV measurement time. After valve deployment, the second LV dP/dt value was paired with the average of three arterial dP/dt values beginning three minutes after valve deployment. Additional information collected from the medical record included: patient demographics, aortic valve area, mean aortic valve gradient, and ejection fraction from the transesophageal echocardiogram prior to surgery. LV and radial arterial pressure measurements were collected from the procedure report and anesthetic record, respectively.

### Data analysis

The collected data were organized offline and summarized using Microsoft Excel. This included patient demographics (age, sex, weight, and height), preoperative ejection fraction, aortic valve area, and mean aortic pressure gradient as determined from the pre-operative transesophageal echocardiogram, LV pressures and dP/dt from the catheterization report and radial arterial pressures and dP/dt values as recorded in the electronic medical record. Pressure gradients and changes after valve deployment were calculated from these primary values.

Demographic variables are presented as mean ± SD. The distribution of all continuous measurements was assessed for normality using the D’Agostino and Pearson test. Most variables were not normally distributed and so all values are expressed as median [95%CI]. Bland-Altman analysis was used to evaluate the agreement of the LV and radial arterial measurements. To further characterize the relationship between the LV and radial dP/dt values as well as assess the acute impact of valve replacement on the primary measurements, we calculated the concordance between left ventricular and radial values following valve deployment as a percent change. The non-parametric Spearman r was calculated to assess correlations between paired measurements and of the difference between paired dP/dt values and other measurements of cardiac function. All statistical analysis used GraphPad Prism version 10.5.0 for Windows (GraphPad Software, San Diego, CA, www.graphpad.com*).*

## Results

Data was available from 84 patients who underwent TAVRs between 07/01/21 and 01/09/24. Three patients did not have data collected after aortic valve deployment or the procedure did not result in valve deployment. All available data from the 84 patients were included in the analysis. Patient demographics are summarized in Table 1. In this study, 54% of the patients were male and 46% were female. Their average age was 76 ± 8 years, average height was 1.70 ± 0.11 m, and average weight was 82.0 ± 20.0 kg. All patients had a baseline transesophageal echocardiogram prior to their procedure. The average ejection fraction (EF) was 57 ± 11%. The average aortic valve area was 0.87 ± 0.29 cm^2^, and the average mean pressure gradient was 39 ± 14 mmHg.

**Table 1:** Patient demographics

Sex	45 Male, 39 Female
Age (years)	76 ± 8.1
Height (m)	1.70 ± 0.11
Weight (kg)	82.0 ± 20.0
Co-existing Disease	(% of total patients)
Hypertension	86
Coronary Arterial Disease	55
Diabetes Mellites (Insulin Dependant)	37 (12)
Hyperdyslipidemia	68
Chronic Obstructive Pulmonary Disease	18
Peripheral Arterial Disease	8
Chronic Renal Disease	20
Baseline Ejection Fraction (%)	57 ± 11
Baseline aortic valve area (cm^2^)	0.87 ± 0.29
Baseline mean gradient (mmHg)	39 ± 14
Post-Deployment mean gradient (mmHg)	4$\:\pm\:$3.1

**Fig. 1:**
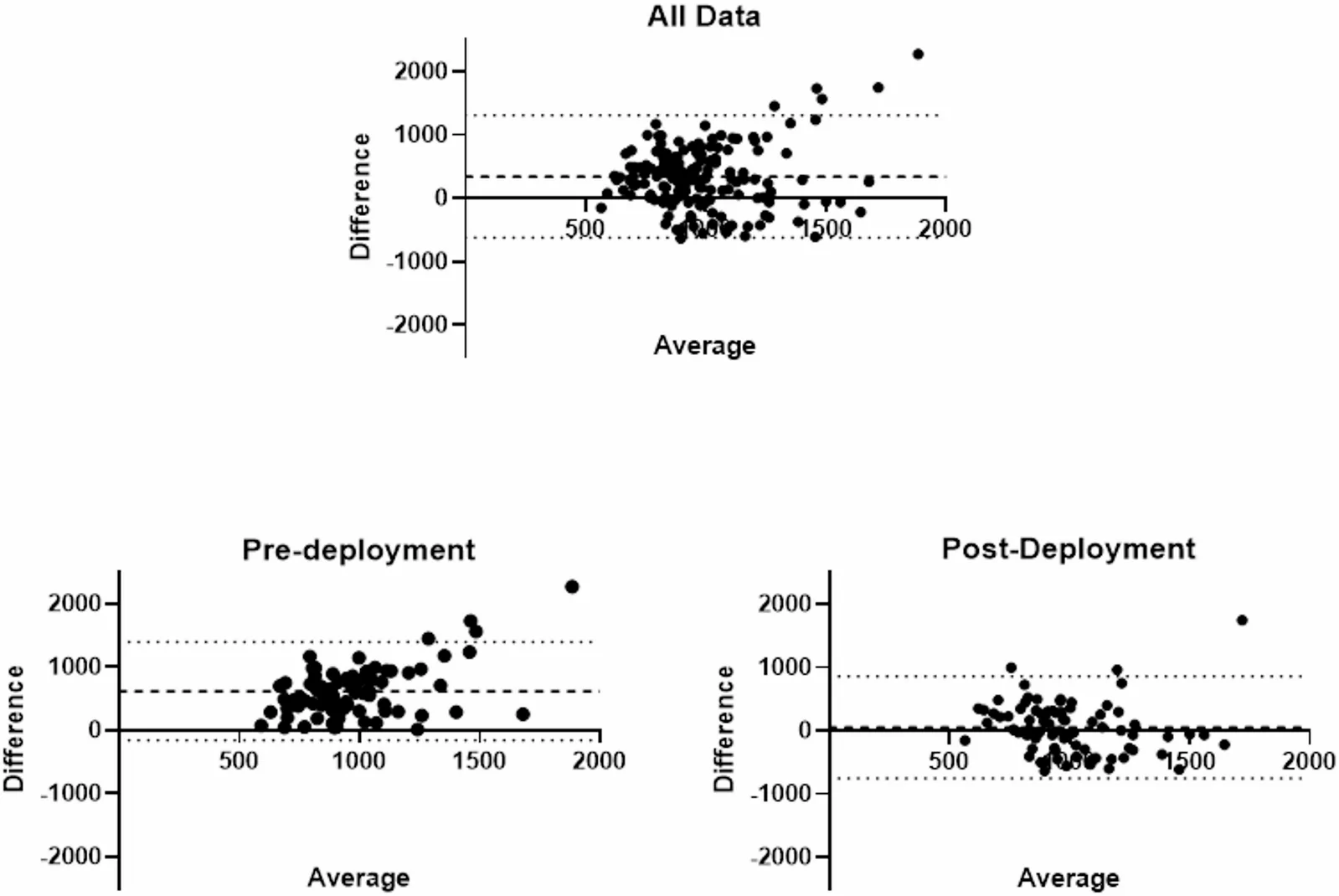
Bland-Altman analysis of LV dP/dt (mmHg/s ) vs. radial arterial dP/dt (mmHg/s). (---) Bias, (∙∙∙) 95% limits of agreement

To evaluate the overall agreement between the radial arterial dP/dt values and the gold standard LV measurements, Bland-Altman analysis of all data points was performed, which demonstrated a bias of 343 ± 493 mmHg/s with 95% limits of agreement from − 623 to 1309 mmHg/s. To assess the possible impact of aortic stenosis, this analysis was repeated before and after valve deployment. Analysis of the values prior to valve deployment demonstrated a bias of 621 ± 396 mmHg/s with 95% limits of agreement from − 155 to 1397 mmHg/s. Following valve deployment, analysis demonstrated a bias of 54 ± 412 mmHg/s with 95% Limits of Agreement from − 754 to 862 mmHg/s (Fig. 1). Although only the post-deployment radial arterial dP/dt values were normally distributed, Lin’s concordance correlation coefficients were calculated and are summarized in Supplementary Table 1.

**Fig. 2:**
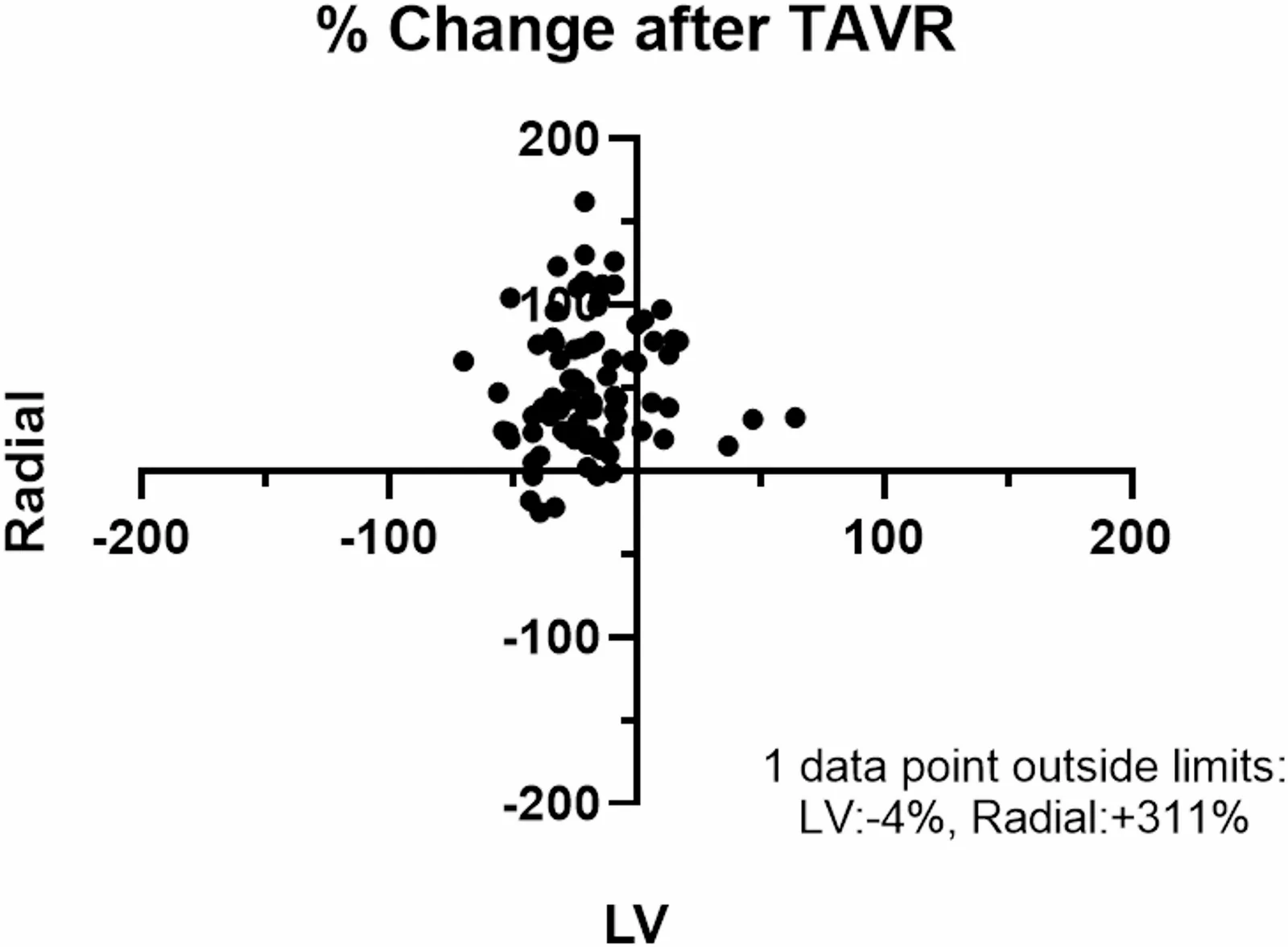
Concordance plot of the %changes in LV and radial arterial dP/dt following TAVR

In patients with both before and after measurements, concordance was graphed as a four-quadrant plot with percent change in LV dP/dt on the x-axis and percent change in radial dP/dt on the y-axis. (Fig. 2). No exclusion zone was used. After valve deployment, 73% of the values were plotted in quadrant one with a decrease in LV dP/dt with an increase in radial dP/dt. Sixteen patients were categorized into quadrant 2 (20%) with a concurrent positive change in LV and radial dP/dt. One patient (1%) had a decrease in LV and increased radial dP/dt (quadrant 3) and five patients (6%) had a concurrent decrease in LV and radial dP/dt (quadrant 4).

**Fig. 3:**
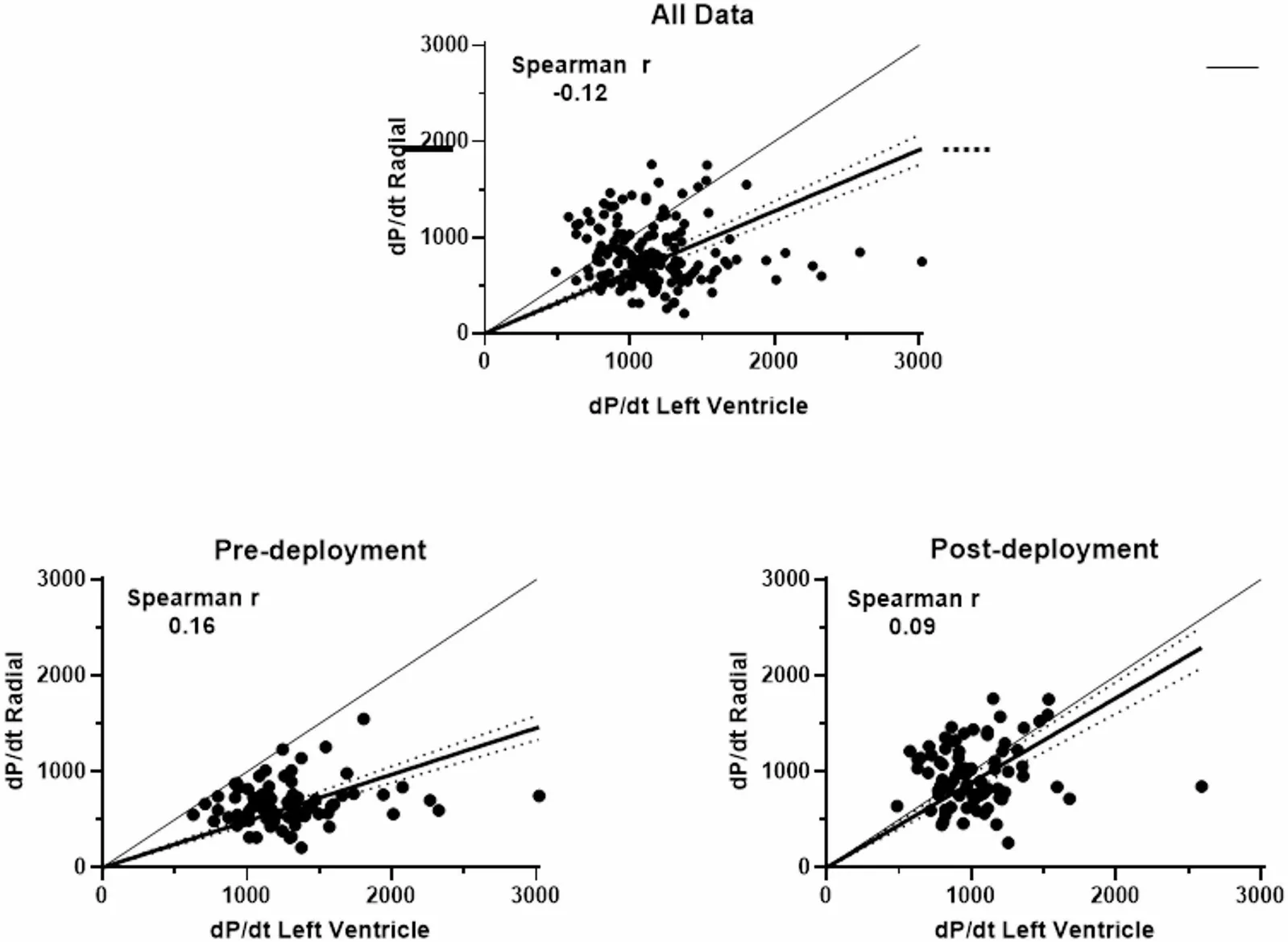
Correlations between LV and radial arterial dP/dt (mmHg/s) measurements for all data and before and following TAVR. Each graph includes a line of identity ( ) and the best-fit regression line ( ) with 95% confidence bands ( ). Note: All graphed regression lines are constrained to the origin

The overall correlation between all paired measurements was poor (Spearman *r* = -0.12 [-0.27,0.04]) and the least squares best-fit line, constrained to the origin to allow comparisons to the expected line of identity, had a slope of 0.64 [0.59, 0.69], R^2^=-0.68. The correlation of all data pairs before valve deployment was *r* = 0.16 [-0.06,0.37] and the best fit line had a slope = 0.48 [0.44, 0.53], R^2^=-0.39. Following valve deployment, the correlation coefficient did not change significantly (*r* = 0.09 [-0.14,0.31), but the relationship was somewhat improved as demonstrated by the line of best fit, which was nearly identical to the line of identity (slope = 0.88 [0.80, 0.97], R^2^=-0.52). (Fig. 3).

**Fig. 4:**
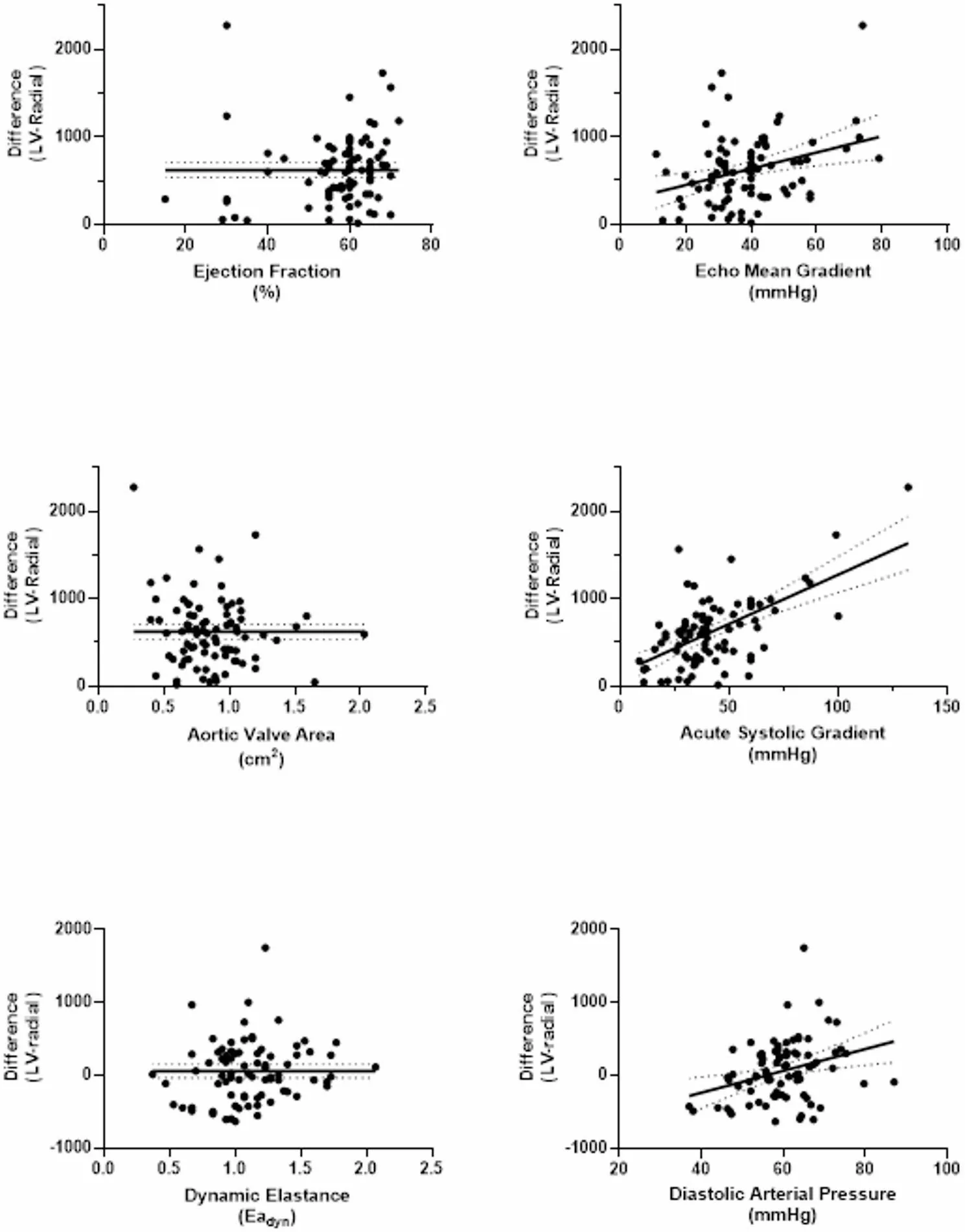
Correlations between the LV and radial arterial dP/dt (mmHg/s) measurement difference and surrogates for contractility and afterload. Each graph includes the best-fit regression line (—) with 95% confidence bands (······)

To determine possible indicators for discrepancies, multiple correlations were evaluated for association with the difference between paired LV and radial arterial dP/dt measurements. Prior to valve deployment, the correlation between the ejection fraction calculated from the pre-operative echocardiogram was poor (Spearman *r* = 0.22 [-0.001, 0.42]) and the least squares best-fit line had a slope that was not significantly different from zero. Similarly, the correlation between the aortic valve area and the difference between measurements was poor (*r* = -0.09 [-0.30,0.14]) and the least squares best-fit line also had a slope that was not significantly different from zero. The correlation for the mean pressure gradient was still poor (*r* = 0.27 [0.05,0.46], but the least squares best-fit line had a slope that was significantly different from zero (slope = 9.3 [3.3, 15.4], R^2^ = 0.10). Lastly, the acute systolic pressure gradient had a weak correlation (*r* = 0.44[0.25,0.60], and a slope that was also significantly different from zero (slope = 11.3 [7.9, 14.6], R^2^ = 0.35). (Fig. 4) After valve deployment, the impact of afterload on the difference between LV and radial measurements was assessed. The correlation for dynamic arterial elastance (Ea_dyn_) was poor (*r* = 0.15 [-0.08, 0.36]) and the least squares best-fit line had a slope that was not significantly different from zero. For diastolic arterial pressure (DAP), the correlation was weak (*r* = 0.31 [0.09, 0.50]) but the least squares best-fit line had a slope that was significantly different from zero (slope = 15 [4.9, 25], R^2^ = 0.10). (Fig. 4).

## Discussion

Myocardial contractility characterizes the ability of the heart to generate the force to propel oxygenated blood through the aortic valve and the systemic circulation. Consequently, its measurement provides therapeutic guidance. LV dP/dt is a gold standard for the assessment of contractility, but it has limitations. It is known to be dependent upon pre-load, but it is characterized as being independent of afterload, because the peak pressure rise occurs before the aortic valve opens [6, 7]. However, early evaluations of the clinical utility of LV dP/dt noted discrepancies that occurred in patients with severe myocardial depression, marked vasodilation and aortic valve stenosis [8–10]. In patients with severe aortic stenosis, the impaired elasticity of the aortic valve is the primary source of afterload, requiring the LV to increase its contractility to maintain cardiac output. This increase in afterload has been suggested by some studies to impact pre-TAVR measurements of dP/dt since a larger pressure gradient is required to force open the stenotic valve, essentially decoupling the left ventricle and afterload [11]. The results of this study support this model and highlight some limitations of radial arterial dP/dt as a measure of contractility.

Direct comparisons between radial and left ventricular measurements of dP/dt in a clinical setting are limited. Data from 12 patients during cardiac catheterization showed poor correlations between the two measurements, but parallel increases during exercise [5]. In animal studies, differences between femoral and LV dP/dt were observed, but the changes in response to altered pre-load and contractility were also parallel in both healthy animals [4] and in those with endotoxin-induced shock, if the intravascular volume was adequate [3]. Direct correlations between LV and femoral arterial dP/dt have been evaluated in a cardiac surgical patient population [12]. There was a good correlation between the two measures, but a significant bias. Femoral arterial dP/dt values tracked LV value changes in response to inotropes and intravascular volume shifts. Other studies have evaluated the correlations of radial arterial dP/dt with other assessments of myocardial contractility and demonstrated agreement is response to changes [13–15]. Radial arterial dP/dt has also been demonstrated to be a useful guide to clinical care focused on optimizing contractility in critically ill patients [16, 17] and in the electrophysiology laboratory during cardiac resynchronization therapy [18].

Prior to TAVR, the observed correlations between LV and radial arterial measurements of dP/dt were poor. Bland-Altman analysis demonstrated substantial bias and wide limits of agreement. In addition, the Spearman correlation coefficient was quite low, suggesting that radial arterial dP/dt does not accurately reflect LV measurements of contractility in the presence of aortic stenosis. This was not unexpected as aortic stenosis is one of the many physiological and pathological conditions that can affect the morphology of the radial arterial pressure waveform [19, 20] In the majority of patients in this study, aortic valve deployment, eliminating the aortic valve stenosis, was associated with a decreased LV dP/dt and increased radial arterial dP/dt. There were corresponding improvements in the LV/radial relationship reflected in the Bland-Altman analysis as minimal bias, but the limits of agreement remained wide. The Spearman correlation also remained poor. However, a positive change in the relation between LV and radial measurements was reflected in the best-fit linear regression line which approached the line of identity.

To provide guidance for clinical situations in which the radial arterial dP/dt measurements should be interpreted more cautiously, evaluations were done to explore potential determinates of the difference between LV and radial arterial measurements both before and after valve deployment. Guided by past studies we evaluated the impact of myocardial depression or global LV function as assessed by the ejection fraction or the mean pressure gradient. To evaluate the impact of afterload we examined the relationship between LV and radial measurements both before and after valve deployment using aortic valve area and the systolic pressure gradient (before) and dynamic arterial elastance and diastolic arterial pressure (after). Before TAVR the mean pressure gradient and the acute systolic gradient correlated with the difference between the LV and radial arterial measurements. After TAVR, there was a correlation between the diastolic arterial pressure and the measurements difference. None of these correlations were robust. The best was the systolic pressure gradient before TAVR which, although statistically significant, was still weak.

This study has limitations. Although larger than previous clinical evaluations, this was a retrospective study of opportunity rather than a prospective clinical trial and consequently, the sample size (*n* = 84) limits the statistical power to accurately identify the relationship between radial arterial and LV dP/dt or the impact of other clinical measurements such as heart rate, preload, blood pressure or the response to inotropic drugs. As observational data, it cannot provide any guidance such as thresholds for interventions. It was also not possible to synchronize the comparisons on a beat-to-beat basis. Measurements were time-synchronized and averaged during periods of transient hemodynamic stability, but the impact of more rapid changes could not be evaluated [21].This lack of beat-to-beat synchronization may have impacted our observed correlations. There is also potential for selection bias. Only 84 of the 454 patients undergoing a TAVR procedure performed in our institution during this period of time included the use of the Acumen IQ Transducer. The reasons guiding this decision are not known, but our review of the use suggests that this is the result of provider preference, not patient characteristics or protocol. The sample size is not large enough to allow any comprehensive characterizations of the patient population to confirm this. Lastly, the clinical care protocol only included two time points to compare, before and after aortic valve deployment. Although the post deployment data imply that radial arterial dP/dt measurement may reflect contractility changes within a given patient, no inotropic agents were given to test this assumption. In addition, this comparison was made immediately following valve replacement, before there was time for any compensatory pathophysiology to change [21]. Expanding the sample size in future studies could clarify whether the observed correlations are clinically meaningful and protocol modifications to include interventions known to induce measurable changes in contractility would provide useful clinical guidance.

Some of the earliest observations regarding LV dP/dt were that it has better clinical utility as a guide to directional changes in contractility for an individual patient, as opposed to providing an absolute measure [9, 10]. The linear relationship between LV and radial measurements following valve deployment suggests that the same is likely true for radial arterial measurements in the absence of aortic stenosis. The absence of any improvement in correlation following valve deployment may be explained by the decrease in vascular compliance which is often seen in elderly patients and in those with cardiovascular disease [22, 23]. While the stenotic aortic valve was replaced, the compliance of the downstream arteries was unchanged. Lastly, the potential presence of concentric LV hypertrophy, a common sequela of aortic stenosis, may have significantly affected LV dP/dt measurements. Chronic increases in LV pressure may lead to LV wall thickening and reduced compliance, resulting in diastolic dysfunction. This limits impact of preload and decreases the LV’s ability to generate the increased pressure expected to be seen during contraction [24].

Efforts continue to search for a truly load-independent assessment of LV contractility with clinical utility [25, 26], but radial arterial dP/dt has many advantages, despite its limitations [17]. The results of this study suggest that concurrent left ventricular and radial arterial measurements of dP/dt are not interchangeable. However, they are consistent with previous studies suggesting that, in the absence of aortic stenosis, radial arterial measurements may reflect changes in contractility within an individual patient. Larger studies are required to explore potential pathologies leading to differences between LV and radial arterial measurements. Study designs focused on confirming the response of radial arterial measurements in response to changes in contractility are recommended.

## Supplementary Information

Supplementary Material 1.

## Data Availability

The data sets used and/or analyzed during the current study are available from the corresponding author on reasonable request.

## Abbreviations

DAP: Diastolic arterial pressure
dP/dt: Change in pressure over time
Ea_dyn_): Dynamic arterial elastance
EF: Ejection fraction
LV: Left ventricular
TAVR: Transcatheter aortic valve replacement
