# Supplementary material for: Radial arterial versus left ventricular dP/dt for assessment of contractility in patients undergoing transcatheter aortic valve replacement: a retrospective observational study
Source: BMC Cardiovasc Disord. 2026 Jun 25;26:798. doi: 10.1186/s12872-026-06159-z (PMC13576193; doi:10.1186/s12872-026-06159-z)
Supplement: Supplementary file 1 [file 12872_2026_6159_MOESM1_ESM.docx]

**Supplementary Table 1**

|  | LV-ALL | Radial-ALL | LV-Pre | Radial-Pre | LV-Post | Radial-Post |
| --- | --- | --- | --- | --- | --- | --- |
| Mean | 1156 | 813 | 1283 | 662 | 1025 | 970 |
| Standard Deviation | 361 | 315 | 373 | 221 | 298 | 322 |
| Test for normal distribution |  |  |  |  |  |  |
| D'Agostino & Pearson test |  |  |  |  |  |  |
| K2 | 74.3 | 16.1 | 44.7 | 25.1 | 53.7 | 2.16 |
| P value | <0.0001 | 0.0003 | <0.0001 | <0.0001 | <0.0001 | 0.3393 |
| Passed normality test? | No | No | No | No | No | Yes |
| Lin's CCC |  | -0.038 |  | 0.054 |  | 0.115 |
| 95% confidence interval |  | -0.138, 0.062 |  | -0.008, 0.116 |  | -0.101, 0.320 |

Average dP/dt vales, results of D'Agostino & Pearson test for normal distribution and Lin’s concordance correlation coefficients for each comparison group.
